# The Chinese version of the Pediatric Quality of Life Inventory™ (PedsQL™) Family Impact Module: cross-cultural adaptation and psychometric evaluation

**DOI:** 10.1186/1477-7525-9-16

**Published:** 2011-03-23

**Authors:** Ruoqing Chen, Yuantao Hao, Lifen Feng, Yingfen Zhang, Zhuoyan Huang

**Affiliations:** 1 School of Public Health, Sun Yat-sen University, Guangzhou 510080, China; 2 Department of Epidemiology, School of Public Health, Fudan University, Shanghai 200032, China; 3 Special Center, the First Affiliated Hospital of Sun Yat-sen University, Guangzhou 510080, China; 4 Section of Otolaryngology Surgery, the First Affiliated Hospital of Sun Yat-sen University, Guangzhou 510080, China

## Abstract

**Background:**

A pediatric chronic health condition not only influences a child's life, but also has impacts on parent health-related quality of life (HRQOL) and family functioning. To provide care and social support to these families, a psychometrically well-developed instrument for measuring these impacts is of great importance. The present study is aimed to evaluate the psychometric properties of the Chinese version of the PedsQL™ Family Impact Module.

**Methods:**

The cross-cultural adaptation of the PedsQL™ Family Impact Module was performed following the PedsQL™ Measurement Model Translation Methodology. The Chinese version of the PedsQL™ Family Impact Module was administered to 136 parents of children with asthma and 264 parents of children with heart disease from four Triple A hospitals. The psychometric properties such as feasibility, internal consistency reliability, item-subscale correlations and construct validity were evaluated.

**Results:**

The percentage of missing item responses was less than 0.1% for both asthma and heart disease sample groups. The Chinese version of the PedsQL™ Family Impact Module showed ceiling effects but had acceptable reliability (Cronbach's Alpha Coefficients were higher than 0.7 in all the subscales except "Daily Activities" in the asthma sample group). There were higher correlation coefficients between items and their hypothesized subscales than those with other subscales. The asthma sample group reported higher parent HRQOL and family functioning than the heart disease sample group. In the heart disease sample group, parents of outpatients reported higher parent HRQOL and family functioning than parents of inpatients. Confirmatory factor analysis showed that the instrument had marginally acceptable construct validity with some Goodness-of-Fit indices not reaching the standard indicating acceptable model fit.

**Conclusions:**

The Chinese version of the PedsQL™ Family Impact Module has adequate psychometric properties and could be used to assess the impacts of pediatric asthma or pediatric heart disease on parent HRQOL and family functioning in China. This instrument should be field tested on parents of children with other chronic medical conditions in other areas. Construct validity tested by confirmatory factor analysis and test-retest reliability should be further assessed.

## Background

The evaluation of pediatric health-related quality of life (HRQOL) is increasingly significant in clinical trials and health care research. In pediatric chronic health conditions, the impact of disease and treatment not only plays an important role in a child's development, but also influences the HRQOL of the parents [1]. Thanks to the advances in medicine and modern technology, the survival rate of children with chronic illness has been increased [2]. However, shortened hospitalizations, long-term consumption of medication and intensive medical treatment in ambulatory settings increase the burdens of the families having pediatric patients with chronic diseases, and affect the family functioning ultimately [2]. Furthermore, the families' capability to deal with the difficulties and uncertainties relevant to their children's diagnosis and treatment could affect the children's quality of life as well [3]. Therefore, the assessment of the impact of pediatric chronic diseases on parental psychosocial status, psychological well-being and functioning is undoubtedly useful, identifying the necessity of family education, psychological intervention and social support for the families in need. This assessment is also valuable for health care professionals and policy makers devoted to improving the HRQOL of children and their parents.

Based on the Chinese population, some studies have been conducted examining the impacts of pediatric chronic diseases such as asthma, congenital heart disease and leukemia on parents. Most of them suggested that the parents of sick children suffered more mental stress and more psychological problems than parents in a control group. Different instruments were used to measure these effects, such as SCL-90 questionnaire, Hospital Anxiety and Depression Scale, Life Event Scale (LES), Way of Dealing with Stress Questionnaire and Life Satisfaction Index A (LSIA) [4-6]. However, none of these studies used a specialized family impact instrument or even a parent HRQOL measurement. Some of them even yielded inconclusive results, decreasing their power to evaluate the impact of the child's health conditions on the family. Thus, the HRQOL of parents can not be completely assessed without a well-developed instrument that specifically measures the impact of pediatric chronic medical conditions on parents and family functioning.

In order to improve the assessment of the impact of pediatric chronic diseases on the parent HRQOL in the context of Chinese culture, we decided to introduce and use the Pediatric Quality of Life Inventory™ (PedsQL™) Family Impact Module (FIM). The FIM is a module of the PedsQL™ Measurement Model which was first developed by James W. Varni et al in 1999. The PedsQL™ Measurement Model is a practical and validated modular instrument for measuring the HRQOL of children aged 2 to 18 [7,8]. It includes a generic core scale, disease-specific modules, family impact module and other condition-specific modules, most of which have demonstrated satisfying psychometric properties [9-11]. The FIM, which was introduced in 2004, could stand alone, or be integrated into the PedsQL™ Measurement Model, allowing an overall assessment of HRQOL of children and parents [12]. The FIM has already been established with adequate reliability and validity for parents of children with complex chronic health conditions, children with cancer, children with sickle cell disease and children with chronic pain [3,12-14]. The PedsQL™ Measurement Model has been widely used in more than 60 countries [8]. Additionally, the PedsQL™ 4.0 generic core scale has been cross-culturally adapted to Chinese and psychometrically evaluated, and the Chinese versions of the Asthma Module and the Cardiac Module are being developed [15].

The objective of the current study was to evaluate the psychometric properties of the Chinese version of the FIM in a pediatric asthma sample and a pediatric heart disease sample. We hypothesized that parents of children with asthma would have higher HRQOL and family functioning than those of children with heart disease based on the extant literature on the association between hospitalization and adverse outcome of disease and the conceptualization of HRQOL as a marker of disease severity [8-10,16-18]. Moreover, we hypothesized that among parents whose children had heart disease, parents of inpatients would report significant differences in HRQOL and family functioning compared with those of outpatients based on previous PedsQL™ FIM findings with other pediatric chronic diseases [3,12].

## Methods

### Participants and Settings

The study was conducted from December, 2008 to June, 2009 in Guangzhou in Guangdong Province of China. Study subjects were recruited from four Triple A hospitals by the convenience sampling method. Triple A hospitals are the best ones in China, which supply high-level medical services and implement high medical education and research tasks. Subjects were approached with the permission of the doctors if: 1) they were the parents of a child, aged 2 to 18, who was an inpatient or an outpatient with asthma, or 2) they were the parents of a child, aged 2 to 18, who was an inpatient or an outpatient with heart disease. The pediatric patients were diagnosed conforming to the national standards for asthma or heart disease diagnosis of China. Heart disease was categorized as follows: 1) congenital heart disease, including aortic valve stenosis, atrial septal defect, patent ductus arteriosus, Tetralogy of Fallot, pulmonary stenosis, complex congenital heart disease and others, or 2) acquired heart disease, including arrhythmia, cardiomyopathy, myocarditis, rheumatic heart disease, infective endocarditis, Kawasaki disease and others. Inpatient was defined as a child who was hospitalized for required treatment. Outpatient was defined as a child who only went to the outpatient department for subsequent visits. Parents were excluded from the study if they were illiterate, reluctant to participate, or their children had other chronic illnesses.

### Instrument

#### PedsQL™ Family Impact Module

The FIM was developed as a parent-reported instrument to measure the impact of pediatric chronic health condition on parent HRQOL and family functioning. This 36-item instrument consists of 8 subscales: Physical Functioning (6 items), Emotional Functioning (5 items), Social Functioning (4 items), Cognitive Functioning (5 items), Communication (3 items), Worry (5 items), Daily Activities (3 items) and Family Relationships (5 items). The former 6 subscales measure parent self-reported functioning, while the latter 2 subscales measure parent-reported family functioning. Each item has five Likert response options which are 0 (never a problem), 1 (almost never a problem), 2 (sometimes a problem), 3 (often a problem) and 4 (almost always a problem). Items are then linearly transformed to a 0-100 scale (0 = 100, 1 = 75, 2 = 50, 3 = 25, 4 = 0), so that higher scores indicate better HRQOL (less negative impact). The subscale scores are computed as the sum of the items divided by the number of items answered within a particular subscale. If over 50% of the items in a subscale are missing, the subscale score is not computed.

Three types of summary scores can be obtained in the FIM: 1) the Total Score is calculated as the sum of all 36 items divided by the number of items answered; 2) the Parent HRQOL Summary Score is calculated as the sum of the 20 items of Physical, Emotional, Social, Cognitive Functioning subscales divided by the number of items answered; 3) the Family Functioning Summary Score is calculated as the sum of the 8 items of Daily Activities and Family Relationships subscales divided by the number of items answered.

#### PedsQL™ Family Information Form

The PedsQL™ Family Information Form was also developed by James W. Varni et al. It has been cross-culturally adapted into Chinese, and contains demographic information including the child's date of birth, gender, disease duration, and the parent's marital status, occupation, level of family income, and method of payment for the child's medical care.

### Cross-cultural adaptation

The aim of the linguistic validation of the FIM was to produce a Chinese version which could be conceptually equivalent to the original American English version [19].

The linguistic validation was conducted following the PedsQL™ Measurement Model Translation Methodology and consisted of 4 steps: forward translation, backward translation, preliminary test and field test [19].

The forward translation from English to Chinese was performed by a pediatrician and a medical English teacher independently, both of whom were fluent users of English. The two drafts were then discussed by a multidisciplinary team which consisted of a pediatrician, a nurse, a health services researcher, and the project manager who was also a statistician. They compared the drafts and agreed on a single reconciled Chinese version to make a combined version, the meanings of which were equivalent to the original one.

The backward translation from the first Chinese version to English was performed by a bilingual pediatrician who was a native Chinese speaker but working in the United States and fluent in English. The translator had no access to the original version of the FIM. The backward version was compared with the original one by the multidisciplinary team. If the team detected any inaccuracy or disaccord, they rectified the instructions and items to assure semantic and conceptual equivalence. The second Chinese version was then yielded.

The second Chinese version was preliminarily tested on a panel of 20 parents. This test was carried out through face-to-face interviews during which the interviewees were free to ask any questions in terms of the contents of the questionnaire or the acceptance of the translation. They were also encouraged to suggest solutions to the identified problems. After the revision of the second version, the Chinese version of the FIM was finalized and to be field-tested in the current study.

The reports of all the steps in the translation process were sent to and accepted by the Mapi Research Institute in Lyon, France, on behalf of Dr. James W. Varni, the copyright owner of the PedsQL™.

### Data collection

The investigation was performed by five undergraduate students majoring in Preventive Medicine and three nurses. All of them were trained by the project manager in order to guarantee the quality of the investigation. The parents were asked to fill out the FIM and the PedsQL™ Family Information Form by means of self-administration during their children's hospitalization or outpatient department visit. The investigators assisted the completion of the questionnaires in case the parents had problems of semantics or conceptual understanding. They were also responsible for collecting the questionnaires and checking for any missing data or logical mistakes. The Ethics Committee of the School of Public Health, Sun Yat-sen University approved the study. Written informed consent forms were obtained from the parents.

### Statistical analysis

Descriptive analysis was used for reporting the demographic characteristics of the parents and children. Continuous variables were presented as median, upper quartile and lower quartile as they followed skewed distributions. Categorical variables were presented as observed frequencies and proportions.

The response rate was calculated as the number of subjects in the analysis divided by the number of subjects approached for the study.

The feasibility of the FIM was assessed by analyzing the percentage of missing item responses and the average completion time.

The presence of floor and ceiling effects (>25% of the respondents have the minimum and/or maximum score) was assessed for the subscale scores and summary scores [20].

Internal consistency reliability was determined using Cronbach's Alpha Coefficient for the subscale scores and summary scores. Values greater than 0.70 were considered acceptable for comparing different groups [21].

Item-subscale correlations were assessed using multitrait scaling analysis. Spearman's rank correlation coefficients were calculated in the multitrait scaling analysis. Good scaling success was supported if the correlations between each item and its hypothesized subscale were stronger than those between the item and other subscales.

Construct validity was evaluated by means of the known-groups method by which the differences of the subscale scores and summary scores across groups could be detected. The Wilcoxon Rank-Sum Test was used to compare 1) parents of children with asthma versus those of children with heart disease, and 2) parents of inpatients versus those of outpatients among parents whose children had heart disease.

Construct validity was further assessed by Confirmatory Factor Analysis (CFA). The aim of CFA was to test the hypothesis that there existed a relationship between the observed variables (items) and their underlying latent constructs (subscales). Model adequacy was evaluated by χ² tests. Since χ² test was sensitive to sample size, χ²/df ratios were also calculated. A χ²/df ratio value of 5.00 or lower indicated adequate model fit [22]. The Comparative Fit Index (CFI), Adjusted Goodness of Fit Index (AGFI), Non-Normed Fit Index (NNFI) and Root Mean Square Error of Approximation (RMSEA) were used as the main Goodness-of-Fit indices. The values of CFI, AGFI, NNFI and RMSEA were in the range of 0 to 1. For both CFI and NNFI, a value of 0.9 or greater was considered as a good degree of "fit" for the model in question [23]. An AGFI value of 0.85 or greater indicated acceptable model fit which could also be demonstrated by a RMSEA value of 0.08 or less [24,25]. The premeditated eight-factor model was specified for the CFA analysis in the current study.

All the analyses were conducted using SPSS 17.0 and LISREL 8.70 for Windows.

## Results

### Sample Characteristics, Response Rate and Feasibility

There were 139 parents of children with asthma and 280 parents of children with heart disease approached for the study. In the asthma sample group, 136 completed the questionnaire except 3 participants who answered less than 50% of the items. In the heart disease sample group, 264 completed the questionnaire, 8 refused to participate since they were in a rush or unwilling to do it, and 8 finished only the Family Information Form but not the FIM. Thus, the response rates were 97.84% and 94.29% respectively. The percentage of missing item responses for the heart disease sample was 0.07%, but there was no missing item response in the asthma sample group. The average completion time was 5 to 8 minutes. Table 1 displays the descriptive analysis of the demographic characteristics of the whole sample. More than half of the subjects were mothers in both groups. On the item "Level of Family Income", over 60% of the asthma sample group reported "intermediate mid", while over 50% of the heart disease sample group reported "intermediate low to low". On the item "Method of Payment for the Child's Medical Care", more than 33% of the heart disease sample group used "rural cooperative medical service", but only 2% of the asthma sample group used it. In addition, over 15% of parents of patients with heart disease reported "severe" disease status of their children, but the percentage was less than 5% in the asthma sample group.

**Table 1:** Demographic Characteristics of the Sample

Demographic Characteristics	Parents of Asthma children (N = 136)		Parents of Heart Disease Children (N = 264)	
	N	%	N	%
**Characteristics of Parents**				
**Relationship to Patient**				
Father	18	13.24	97	36.74
Mother	105	77.21	156	59.09
Grandfather	0	0.00	5	1.89
Grandmother	12	8.82	3	1.14
Others	1	0.74	3	1.14
**Level of Family Income**				
High	1	0.74	0	0.00
Intermediate high	17	12.50	8	3.03
Intermediate mid	92	67.65	104	39.39
Intermediate low	22	16.18	84	31.82
Low	4	2.94	68	25.76
**Method of Payment for the Child's Medical Care**				
Free medical service	14	10.29	6	2.27
Medical insurance	43	31.62	52	19.70
Rural cooperative medical service	3	2.21	89	33.71
Self-paying	75	55.15	114	43.18
Others	1	0.74	3	1.14
				
**Characteristics of Children**				
**Ages (years)**				
2~4	59	43.38	116	43.94
5~7	42	30.88	61	23.11
8~2	31	22.79	43	16.29
13~18	4	2.94	44	16.67
**Gender**				
Male	93	68.38	150	56.82
Female	43	31.62	114	43.18
**Groups**				
Inpatient	1	0.74	207	78.41
Outpatient	135	99.26	57	21.59
**Disease Duration (years)**				
<2	51	37.50	74	28.03
2~	54	39.71	82	31.06
≥4	31	22.79	108	40.91
**Disease Status**				
Mild	91	66.91	156	59.09
Moderate	35	25.74	66	25.00
Severe	6	4.41	42	15.91
Not reported	4	2.94	0	0.00

### Cross-cultural adaptation

The cross-cultural adaptation was performed not only following the PedsQL™ Measurement Model Translation Methodology but also fully taking into account the Chinese culture and national conditions. During the preliminary test, the interviewees reported that they had no difficulties understanding the questionnaire. Although most of them understood the importance of the research, several interviewees did not enjoy answering the questions since the items with words of negative meanings, e.g. tired, sad, frustrated and isolated, made them feel uncomfortable.

### Modes of administration

In the current study, the face-to-face interview was determined as another mode of administration besides the self-administration, and about 65% of the subjects completed the questionnaire in the interview mode. This option was made to improve the quality and quantity of completed questionnaires. The face-to-face interviews were conducted by the investigators if: 1) the parent was unable to read more than 20% of the items, or 2) the parent had limited time or was unable to fill out the questionnaire because he/she needed to take care of the child or other stuff.

### Subscale response descriptives

Table 2 displays median, upper and lower quartiles, floor and ceiling effects on each subscale score and summary scores of the FIM for the asthma sample group and the heart disease sample group. Test of Normality (Kolmogorov-Smirnov Test) indicated non-normal distributions of the item responses in the FIM for both groups. Most of the skewness and kurtosis values of subscales were below 0, further demonstrating skewed distributions. The FIM showed ceiling effects but no floor effect in all the subscale scores and summary scores for both groups.

**Table 2:** Subscale Descriptives and Construct Validity of the FIM in Parents of Children with asthma and Parents of children with Heart Disease

	Parents of Asthma children			Parents of Heart Disease Children			*Z*	*p*
Scale	N	Median (Q_L_,Q_U_)	%Floor/%Ceiling	N	Median (Q_L_,Q_U_)	%Floor/%Ceiling		
**Total Score**	136	79.17 (65.28, 89.58)	2.66/50.76	264	71.88 (57.12, 86.81)	3.45/37.71	-2.757	0.006
**Parent HRQOL Summary Score**	136	78.13 (64.06, 92.50)	1.95/48.79	264	73.75 (57.81, 88.75)	2.44/38.66	-1.661	0.097
Physical Functioning	136	75.00 (58.33, 94.79)	2.08/44.49	264	75.00 (58.33, 91.67)	2.02/38.26	-0.626	0.531
Emotional Functioning	136	80.00 (60.00, 95.00)	1.62/48.53	264	75.00 (55.00, 90.00)	2.80/37.88	-2.409	0.016
Social Functioning	136	81.25 (62.50, 100.00)	3.86/55.14	264	75.00 (56.25, 93.75)	4.37/39.77	-2.594	0.009
Cognitive Functioning	136	80.00 (60.00, 100.00)	0.59/49.12	264	75.00 (60.00, 95.00)	1.06/39.02	-0.926	0.355
Communication	136	100.00 (75.00, 100.00)	0.26/69.36	264	75.00 (58.33, 100.00)	2.78/41.29	-5.897	<0.001
Worry	136	70.00 (46.25, 83.75)	8.09/39.85	264	60.00 (45.00, 78.75)	9.32/25.68	-2.767	0.006
**Family Functioning Summary Score**	136	82.81 (71.88, 93.75)	1.93/55.51	264	75.00 (59.38, 93.75)	2.56/41.52	-2.536	0.011
Daily Activities	136	66.67 (50.00, 83.33)	4.41/39.71	264	66.67 (50.00, 91.67)	3.28/31.94	-0.466	0.641
Family Relationships	136	95.00 (75.00, 100.00)	0.44/65.00	264	80.00 (65.00, 100.00)	2.12/47.27	-3.498	<0.001

Q_L _= lower quartile; Q_U _= upper quartile; %Floor/%Ceiling = percentage of scores at the extremes of the scaling range

### Internal consistency reliability

Internal consistency reliability Cronbach's Alpha Coefficients for the FIM are presented in Table 3. For the total sample, the asthma sample and the heart disease sample, the coefficients of all the subscale scores (except "Daily Activities" in the asthma sample group) and summary scores were higher than 0.70.

**Table 3:** Internal Consistency Reliability of the FIM in Parents of Children with Asthma and Parents of Children with Heart Disease

Scale	Total	Parents of Asthma children	Parents of Heart Disease Children
**Total Score**	0.97	0.96	0.97
**Parent HRQOL Summary Score**	0.95	0.94	0.96
Physical Functioning	0.89	0.88	0.89
Emotional Functioning	0.89	0.90	0.89
Social Functioning	0.83	0.76	0.85
Cognitive Functioning	0.90	0.87	0.92
Communication	0.83	0.80	0.82
Worry	0.84	0.79	0.87
**Family Functioning Summary Score**	0.89	0.86	0.90
Daily Activities	0.80	0.63	0.87
Family Relationships	0.93	0.92	0.93

Values denote Cronbach's Alpha Coefficient

### Item-subscale correlations

Spearman's rank correlation coefficients between items and subscale scores are shown in Table 4. The results showed that except the one between the item "I feel isolated from others" and the subscale "Social Functioning" in the asthma sample group, the Spearman's rank correlation coefficients between items and their hypothesized subscales were mostly significantly higher than those with other subscales.

**Table 4:** Item-subscale correlations of the FIM in Parents of Children with asthma and Parents of Children with Heart Disease

Scale	Physical Functioning	Emotional Functioning	Social Functioning	Cognitive Functioning	Communication	Worry	Daily Activities	Family Relationships
**Physical Functioning**								
...feel tired during the day	**0.811**	0.436	0.595	0.363	0.293	0.417	0.371	0.259
	***0.840***	*0.626*	*0.680*	*0.590*	*0.572*	*0.526*	*0.535*	*0.431*
...feel tired when I wake up in the morning	**0.856**	0.511	0.594	0.536	0.382	0.470	0.424	0.420
	***0.842***	*0.573*	*0.620*	*0.583*	*0.485*	*0.487*	*0.546*	*0.365*
...feel too tired to do the things I like to do	**0.817**	0.578	0.639	0.506	0.427	0.429	0.389	0.424
	***0.872***	*0.681*	*0.686*	*0.639*	*0.616*	*0.538*	*0.600*	*0.496*
...get headaches	**0.730**	0.584	0.473	0.452	0.494	0.455	0.433	0.475
	***0.825***	*0.643*	*0.650*	*0.601*	*0.542*	*0.460*	*0.473*	*0.419*
...feel physically weak	**0.834**	0.570	0.579	0.546	0.478	0.480	0.421	0.438
	***0.847***	*0.648*	*0.648*	*0.598*	*0.544*	*0.488*	*0.549*	*0.494*
...feel sick to my stomach	**0.610**	0.506	0.433	0.456	0.513	0.295	0.388	0.465
	***0.602***	*0.523*	*0.467*	*0.535*	*0.482*	*0.419*	*0.490*	*0.497*
**Emotional Functioning**								
...feel anxious	0.626	**0.828**	0.528	0.573	0.474	0.509	0.484	0.476
	*0.670*	***0.792***	*0.634*	*0.581*	*0.570*	*0.476*	*0.522*	*0.449*
...feel sad	0.565	**0.880**	0.560	0.526	0.607	0.536	0.420	0.557
	*0.624*	***0.869***	*0.582*	*0.554*	*0.603*	*0.540*	*0.528*	*0.464*
...feel angry	0.466	**0.819**	0.523	0.517	0.529	0.455	0.393	0.490
	*0.524*	***0.743***	*0.459*	*0.498*	*0.455*	*0.406*	*0.408*	*0.368*
...feel frustrated	0.550	**0.811**	0.532	0.524	0.564	0.555	0.391	0.450
	*0.646*	***0.886***	*0.648*	*0.671*	*0.658*	*0.585*	*0.573*	*0.475*
...feel helpless or hopeless	0.528	**0.823**	0.574	0.554	0.610	0.587	0.448	0.554
	*0.679*	***0.842***	*0.713*	*0.691*	*0.671*	*0.559*	*0.596*	*0.521*
**Social Functioning**								
...feel isolated from others	0.533	0.598	**0.636**	0.548	0.684	0.515	0.447	0.528
	*0.624*	*0.708*	***0.740***	*0.659*	*0.669*	*0.464*	*0.568*	*0.545*
...trouble getting support from others	0.552	0.595	**0.753**	0.547	0.619	0.460	0.475	0.510
	*0.635*	*0.574*	***0.788***	*0.586*	*0.542*	*0.376*	*0.520*	*0.431*
...hard to find time for social activities	0.517	0.404	**0.832**	0.444	0.311	0.407	0.492	0.266
	*0.627*	*0.548*	***0.880***	*0.612*	*0.525*	*0.449*	*0.594*	*0.380*
...enough energy for social activities	0.605	0.537	**0.826**	0.466	0.526	0.535	0.469	0.421
	*0.712*	*0.647*	***0.894***	*0.667*	*0.608*	*0.520*	*0.651*	*0.508*
**Cognitive Functioning**								
...hard to keep my attention on things	0.625	0.575	0.610	**0.753**	0.472	0.513	0.443	0.407
	*0.703*	*0.637*	*0.728*	***0.851***	*0.599*	*0.529*	*0.613*	*0.499*
...hard to remember what people tell me	0.390	0.489	0.431	**0.819**	0.352	0.280	0.254	0.202
	*0.609*	*0.616*	*0.607*	***0.884***	*0.563*	*0.459*	*0.542*	*0.548*
...hard to remember what I just heard	0.391	0.445	0.421	**0.817**	0.409	0.288	0.312	0.274
	*0.627*	*0.620*	*0.654*	***0.897***	*0.601*	*0.447*	*0.589*	*0.471*
...hard to think quickly	0.542	0.529	0.520	**0.829**	0.385	0.388	0.372	0.365
	*0.619*	*0.648*	*0.656*	***0.875***	*0.653*	*0.511*	*0.623*	*0.557*
...trouble remembering what I was just thinking	0.511	0.564	0.492	**0.815**	0.471	0.416	0.326	0.376
	*0.604*	*0.598*	*0.601*	***0.850***	*0.594*	*0.456*	*0.586*	*0.501*
**Communication**								
...others do not understand my family's situation	0.462	0.566	0.529	0.504	**0.890**	0.485	0.455	0.566
	*0.615*	*0.639*	*0.665*	*0.691*	***0.863***	*0.564*	*0.589*	*0.604*
...hard to talk about my child's health with others	0.442	0.534	0.495	0.413	**0.828**	0.530	0.457	0.574
	*0.553*	*0.604*	*0.559*	*0.531*	***0.867***	*0.528*	*0.494*	*0.488*
...hard to tell doctors and nurses how I feel	0.371	0.497	0.484	0.480	**0.725**	0.507	0.337	0.535
	*0.572*	*0.597*	*0.565*	*0.598*	***0.848***	*0.534*	*0.550*	*0.590*
**Worry**								
...my child's medical treatments are working	0.484	0.464	0.493	0.392	0.421	**0.817**	0.406	0.345
	*0.524*	*0.529*	*0.452*	*0.497*	*0.500*	***0.842***	*0.437*	*0.305*
...side effects of my child's medical treatments	0.349	0.415	0.391	0.282	0.321	**0.782**	0.376	0.318
	*0.506*	*0.491*	*0.429*	*0.463*	*0.469*	***0.863***	*0.501*	*0.335*
...how others will react to my child's condition	0.346	0.512	0.393	0.312	0.568	**0.652**	0.471	0.507
	*0.462*	*0.495*	*0.427*	*0.424*	*0.574*	***0.790***	*0.506*	*0.352*
...my child's illness affects other family members	0.419	0.489	0.462	0.423	0.581	**0.601**	0.434	0.479
	*0.524*	*0.509*	*0.503*	*0.527*	*0.545*	***0.717***	*0.593*	*0.437*
...my child's future	0.383	0.436	0.425	0.375	0.461	**0.785**	0.443	0.379
	*0.431*	*0.486*	*0.408*	*0.394*	*0.464*	***0.787***	*0.516*	*0.318*
**Daily Activities**								
...Family activities taking more time and effort	0.309	0.295	0.375	0.222	0.336	0.401	**0.727**	0.313
	*0.539*	*0.533*	*0.596*	*0.573*	*0.545*	*0.642*	***0.864***	*0.437*
...Difficulty finding time to finish household tasks	0.390	0.418	0.426	0.303	0.425	0.450	**0.777**	0.356
	*0.584*	*0.568*	*0.644*	*0.605*	*0.566*	*0.517*	***0.914***	*0.508*
...Feeling too tired to finish household tasks	0.502	0.521	0.586	0.472	0.451	0.509	**0.782**	0.428
	*0.629*	*0.600*	*0.648*	*0.640*	*0.561*	*0.505*	***0.901***	*0.529*
**Family Relationships**								
...Lack of communication between family members	0.480	0.560	0.471	0.381	0.612	0.502	0.468	**0.867**
	*0.506*	*0.509*	*0.487*	*0.539*	*0.545*	*0.373*	*0.533*	***0.872***
...Conflicts between family members	0.409	0.553	0.402	0.347	0.604	0.413	0.351	**0.885**
	*0.432*	*0.422*	*0.451*	*0.477*	*0.504*	*0.374*	*0.443*	***0.881***
...Difficulty making decisions together as a family	0.483	0.518	0.453	0.361	0.605	0.472	0.421	**0.837**
	*0.484*	*0.507*	*0.517*	*0.565*	*0.592*	*0.413*	*0.526*	***0.892***
...Difficulty solving family problems together	0.512	0.577	0.479	0.443	0.622	0.499	0.440	**0.828**
	*0.489*	*0.489*	*0.475*	*0.547*	*0.592*	*0.377*	*0.528*	***0.893***
...Stress or tension between family members	0.437	0.442	0.466	0.329	0.530	0.453	0.395	**0.816**
	*0.464*	*0.491*	*0.463*	*0.490*	*0.589*	*0.402*	*0.444*	***0.867***

Values denote Spearman's rank correlation coefficients (*p < 0.01*)

Bold = Spearman's rank correlation coefficients between items and their hypothesized subscales

In each cell, the asthma sample coefficients are shown above and the heart disease sample coefficients are shown below in italics

### Construct validity

Construct validity of the FIM assessed by the known-groups method is presented in Table 2 and Table 5. The asthma sample group reported significantly higher total score, family summary score, and most of the subscale scores than the heart disease sample group (*p < 0.05*). Furthermore, when we looked for differences in scores between these two groups, excluding inpatient cases, we only found a significant difference in the subscale "Communication". Among the heart disease sample group, the parents of outpatients reported significantly higher values in the total score, summary scores and all the subscales except "Daily Activities" than the parents of inpatients (*p < 0.05*).

**Table 5:** Construct Validity of the FIM in Parents of Children with Heart Disease: Comparison between Inpatient and Outpatient Samples

**Scale**	**Inpatient Sample**		**Outpatient Sample**		***Z***	***p***
	**N**	**Median (Q_L_,Q_U_)**	**N**	**Median (Q_L_,Q_U_)**		
**Total Score**	207	68.75 (54.86, 86.11)	57	76.39 (65.28, 91.32)	-2.880	0.004
**Parent HRQOL Summary Score**	207	71.25 (55.00, 88.75)	57	78.75 (67.50, 93.13)	-2.549	0.011
Physical Functioning	207	70.83 (54.17, 91.67)	57	75.00 (66.67, 91.67)	-2.355	0.019
Emotional Functioning	207	70.00 (55.00, 90.00)	57	75.00 (65.00, 95.00)	-2.023	0.043
Social Functioning	207	75.00 (50.00, 87.50)	57	75.00 (62.50, 100.00)	-2.183	0.029
Cognitive Functioning	207	75.00 (55.00, 95.00)	57	80.00 (75.00, 100.00)	-2.455	0.014
Communication	207	75.00 (58.33, 100.00)	57	83.33 (70.83, 100.00)	-2.542	0.011
Worry	207	55.00 (40.00, 75.00)	57	70.00 (52.50, 85.00)	-2.744	0.006
**Family Functioning Summary Score**	207	71.88 (56.25, 93.75)	57	84.38 (68.75, 95.31)	-2.723	0.006
Daily Activities	207	66.67 (50.00, 83.33)	57	75.00 (58.33, 95.83)	-1.800	0.072
Family Relationships	207	75.00 (60.00, 100.00)	57	95.00 (75.00, 100.00)	-2.924	0.003

Q_L _= lower quartile; Q_U _= upper quartile

Construct validity of the FIM was also determined by CFA. The Goodness-of-Fit results of the eight-factor model based on the original scaling structure are presented in Table 6. For both groups, CFI values and NNFI values were greater than 0.90. But RMSEA values were a little higher than 0.08, and AGFI values did not reach the value of 0.85.

**Table 6:** Goodness-of-fit Indices Values for the Eight-factor Model

Samples	χ²	df	χ²/df	RMSEA (95%CI)	CFI	NNFI	AGFI
**Parents of Asthma children**	1181.59	566	2.09	0.086 (0.079~0.094)	0.96	0.95	0.63
**Parents of Heart Disease Children**	1532.33	566	2.71	0.083 (0.078~0.088)	0.97	0.97	0.70

df = degree of freedom; RMSEA = Root Mean Square Error of Approximation; CI = Confidence Interval; CFI = Comparative Fit Index; NNFI = Non-Normed Fit Index; AGFI = Adjusted Goodness of Fit Index.

## Discussion

The current study presents the feasibility, reliability and validity of the Chinese version of the FIM. This is also the first report of psychometric properties of the FIM in a pediatric asthma sample and a pediatric heart disease sample. The FIM is a well-developed HRQOL measurement and has been adapted for use in other countries. The development of the Chinese version of the FIM will not only fill the gap in the parent HRQOL assessment in China, but also make it possible to compare impacts of pediatric chronic health conditions on parent HRQOL and family functioning across countries.

In the process of cross-cultural adaptation, the recruitment of translators and specialists in the multidisciplinary team was emphasized since their opinions and suggestions with respect to the development of the Chinese version carried weight. The PedsQL™ Measurement Model Translation Methodology was strictly followed to finalize the Chinese version.

Internal consistency reliability was examined using Cronbach's Alpha Coefficients. All the coefficients (except the one of "Daily Activities" in the asthma sample) exceeded the recommended standard of 0.70 for group comparison, indicating acceptable reliability of the FIM. These findings were consistent with those of the prior studies [12,14]. The Cronbach's Alpha Coefficient of "Daily Activities" in the asthma sample group (0.63) did not achieve the standard value probably because of the small sample size (N = 136).

The Spearman's rank correlation coefficients between items and subscale scores were computed to determine the item-subscale correlations. The correlation coefficient between the item "I feel isolated from others" and its hypothesized subscale "Social Functioning" in the asthma sample group was 0.636. This was not the highest of the coefficients between this item and all the subscales, but the correlation was moderate to strong. Good scaling success was supported because other Spearman's rank correlation coefficients between items and their hypothesized subscales were mostly significantly higher than those with other subscales.

Construct validity was assessed based on the principle that certain specified groups of subjects may be anticipated to score differently from others [26]. The hypothesis was supported: parents of children with asthma had higher HRQOL and family functioning than parents of children with heart disease. There may be several explanations: in the heart disease sample group, the proportion of subjects who came from rural areas was much higher; the percentage of children who had "severe" disease status was greater while the percentage of "mild" disease status was lower; they had a lower level of family income; most of their children were hospitalized for required treatment, which consumed more time and money for the parents. Another hypothesis was verified: in the heart disease sample group, parents of outpatients reported higher HRQOL and family functioning than parents of inpatients. These results were different from those of two prior studies which reported worse parent HRQOL and family functioning in parents of children receiving outpatient treatment compared with those of children receiving inpatient treatment [3,12]. In the current sample, self-paying was the main method of payment for a child's medical care, so parents suffered the impact of financial pressure due to their children's chronic health condition especially when the children required hospitalization. These parents also needed to spend more time accompanying their children and might experience more stress from work and family. Further research will be required to compare the differences of the impacts between inpatient sample and outpatient sample in groups with different cultural backgrounds.

In a previous study, Exploratory Factor Analysis (EFA) was performed to evaluate the construct validity. The analysis found a five-factor model but the factor structure deviated from the theoretical expectation [14]. In the current study, CFA was utilized to determine the construct validity of the FIM. The premeditated eight-factor model demonstrated adequate model fit by χ²/df ratios. CFI and NNFI reached acceptable values. AGFI and RMSEA did not reach the standards indicating acceptable model fit. These implied that the instrument had marginally acceptable construct validity.

Similar to the findings in one prior study, we found ceiling effects but no floor effect in all the subscales of the FIM [14]. This suggested that the instrument might not be sensitive to detect HRQOL improvement in parents who had been doing well but could indicate the HRQOL changes in parents who were experiencing negative impacts from their sick children.

Certain limitations should be considered within this study. The FIM was designed to be self-ministered. However, subjects who had lower level of education or had limited time to fill out the questionnaire were led to administer the instrument in the face-to-face interviews. Previous studies held different views on the impact of modes of administration on the performance of questionnaires [27,28]. Further studies should take modes of administration into account and detect the differences between the interview mode and self-administration mode. In this study, test-retest reliability was not analyzed since the FIM was administered only once during the patient's visit to the outpatient department or the hospitalization. Additionally, the results may not be generalized to other regions. Further studies should be conducted to test the psychometric properties on other samples in other areas.

## Conclusions

The Chinese version of the FIM presents adequate psychometric properties. This suggests that it could be used to assess the impacts of pediatric asthma or pediatric heart disease on parent HRQOL and family functioning in China. The FIM should be field tested on samples of children with other chronic health conditions in other areas, especially the rural areas. Construct validity tested by confirmatory factor analysis and test-retest reliability should be further determined.

## Abbreviations

PedsQL™: Pediatric Quality of Life Inventory™; FIM: Family Impact Module; HRQOL: Health-Related Quality of Life.

## Competing interests

The authors declare that they have no competing interests.

## Authors' contributions

RC conceptualized and designed the study, acquired, analyzed and interpreted the data, and drafted the manuscript. YH conceptualized and designed the study, supervised the data analysis and revised the manuscript. LF conceptualized and designed the study, acquired, analyzed and interpreted the data, and revised the manuscript. YZ and ZH conceptualized and designed the study, acquired the data, and revised the manuscript. All authors read and approved the final manuscript.
